# Can case-based discussions be used for leadership development in medical education? - an exploratory inquiry

**DOI:** 10.15694/mep.2018.0000117.1

**Published:** 2018-06-01

**Authors:** Jamiu O. Busari, Lisa N. Isbouts

**Affiliations:** 1 Maastricht University; 2 Radboud University Medical Centre

**Keywords:** case-based discussion, postgraduate, competency, leadership, medical education

## Abstract

This article was migrated. The article was marked as recommended.

Background:

The organization and funding of health care delivery are undergoing rapid change. As a result, the need for effective and context relevant educational methods to teach physicians leadership skills is growing. The case-based discussion has been proven to be effective in teaching leadership skills. Therefore, we decided to explore the potential value of its application in leadership training programs for residents.

Summary:

We performed a qualitative survey to investigate the views of residents and professionals on the use of case-based discussion for leadership skills development during residency training. The Results showed that there were differences in the quality and methods of teaching amongst teachers in different disciplines. More interactive educational strategies were recommended such as case-based discussion and practice-based education during protected teaching slots. Our findings reflected the challenges associated with finding the perfect moment to initiate formal leadership education in postgraduate medical education.

Conclusion:

The case-based discussion is a potentially helpful method to teach residents leadership skills. In addition, establishing trust between stakeholders in the health system should be a focus of any leadership course. Still, determining the perfect timing to initiate leadership training during residency remains difficult.

## Background

There is a growing need for healthcare leaders who possess the capabilities to deliver care that serves the needs of their communities well. According to Frist (2005), healthcare delivery in the 21 ^st^ century should be consumer driven, patient-centered and provider friendly ( Frist et al. 2005). Frenk et al. (2010) argued in this same light that the education of future physicians should in addition to being informative and formative, also be transformative. Rather than learning to acquire knowledge and skills (educational) or to socialize training around predefined values (developmental) the focus of transformative education is to provide future physicians with the leadership attributes that would enable them to be effective change agents ( Frenk et al. 2010).

Currently, in clinical practice, many clinical disciplines choose and apply their teaching methods differently. In addition to the difference in instructional methods, (i.e., teacher or student-centered) differences exist in the frequency of teaching as well as the level of involvement of trainees in the process. Despite the knowledge and the proven advantages of using adult learning principles in teaching, clinical instructors continue to use classical teacher-centered approaches in practice to teach residents and students. In the ongoing quest for the best way to align the education of physicians in training with the changing needs of in health care systems, it is imperative that the right didactic approach is chosen to teach residents. Since 2008, we have been researching the residents’ needs for leadership education in our institution as well as the various educational strategies to deliver the leadership curriculum we designed following the needs assessment ( Busari et al. 2011). There are various student-centered teaching approaches in the literature that can be used to teach residents. These include the flipped classroom, case-based discussion (CBD), peer teaching and the simulation clinic. In recent studies, we also demonstrated that the flipped classroom was a valuable method to teach certain aspects of leadership to residents ( Lucardie et al. 2017a; Lucardie et al. 2017b). CBD has been proven to have potential in teaching analytical and decision-making skills, and we believe, liked the flipped classroom, that it would be equally beneficial to teach important aspects of management and leadership in residents ( Jerrard 2005). CBD as a teaching method originated from the Harvard Business School and is widely used in many business schools nowadays (Harrison-Walker 2010). While the use of CBD to teach medicine in medical institutions is a recent development, there is a growing body of evidence supporting its effectiveness with positive outcomes in competency-based training ( DeBehnke et al. 1995; Vertrees et al. 2013; Tolchin et al. 2013). In line with our continued goal to develop a leadership-training curriculum for residents, we decided to explore the views of stakeholders on the suitability of CBD as an instructional method to teach negotiation skills. This topic was one of 10 themes that we identified from a needs assessment study on leadership development among Dutch medical residents ( Busari 2012; Brouns et al. 2011; Berkenbosch et al. 2014).

## Methods

We chose a qualitative approach for this study and used semi-structured interviews as our intervention. The focus of the discussions was on how respondents perceived the educational needs of residents and the role of CBD as an educational intervention. We conducted the interviews in September 2015 in a large peripheral hospital in the south of the Netherlands with more than 150 residents. Eleven respondents participated in the survey and were selected using convenience sampling. Six of the respondents were medical residents from two different disciplines (emergency medicine and pediatrics), and five professionals included a medical education administrator, two medical specialists holding managerial positions (pediatrics and surgery) and two hospital administrators. We requested and obtained ethical approval from the ethical review board of the Zuyderland Medical Center (METC nr: 15-N-02).

Interpretative Phenomenological Analysis (IPA) was used to analyze our data because our objective was to describe respondents’ perceptions of their experiences and explore how they derived meaning from them ( Berkenbosch et al. 2013). A grounded theory approach was not suitable as we were interested in generating new theoretical concepts. Each interview was recorded, transcribed and thematic analysis was used to inductively analyze content. Excerpts revealing participants’ perceptions of CBD were identified and coded and later grouped into two themes. These themes were 1. The value of CBD in medical education and 2. The value of CBD for leadership development.

## Results

Our study revealed that all of the residents had a good knowledge of different teaching methods, although none of them had experienced a CBD teaching session before. While most of the residents we surveyed knew about case-based discussions (CBD), not all of the professionals we interviewed did. Asked if they prepared before attending teaching session, the residents reported that they did not prepare for the encounters. The reason they gave was that of a perceived general lack of time due to clinical responsibilities. The professionals also recognized this limitation.

### The potential value of CBD in medical education

Most of the residents interviewed, saw room for improvement in the structure of the teaching they received. There was a general desire for more interactive, practice- and case-based teaching sessions to improve the perceived learning experience. Interestingly, all of the professionals felt that once explained, CBD was an efficient educational method for residents. One of the residents also mentioned that there were too many teaching sessions in her department. These sessions were often short and some of them obligatory even when they were off-duty. Asked about potential strategies for improving the teaching and learning experience, the respondents felt that the introduction of a new teaching method could be useful. However, they were hesitant to participate in efforts to help implement such sessions due to the general sense of a shortage of time and the feeling that their efforts would not significantly impact a change in the educational process.

### The potential value of CBD for leadership development

On current teaching approaches, the professionals were critical in reviewing their educational qualities. They stated that the quality of the teaching abilities varied widely, despite the fact that they had all participated in ‘teach the teacher’ training. One professional and most of the residents stated that often, clinical teachers know what to teach, but cannot often execute it. As a result, they tend to resort to teaching methods that they are familiar from their training, i.e., teacher-centered teaching styles. Despite this, residents still perceived instructors to be motivated to teach stating “ *you feel the teachers’ passion for the profession*” and that supervisors were often open for questions and discussions. These observations also reflect the importance and need for well-defined formats in instructional approach. In response, the professionals suggested the use of more interdisciplinary, case-based and interactive teaching sessions. Their arguments corroborated some of the recommendations the residents gave for more structured teaching formats and protected time for teaching. As one of the professionals summarized: *‘The future of resident education should be more informal, open and spontaneous with short individual learning moments on the work floor and classic set teaching moments where residents cannot be interrupted or punished for, by having to work extra hours after the teaching sessions.’*

On educating residents to be future physician leaders, the specialists stated that an understanding of the health care system was a crucial requirement. Furthermore, they felt that it would be difficult to apply any leadership skills learned, without mutual trust and the willingness to understand the interests of the different stakeholders in the healthcare system. Two of the professionals felt that the ongoing discourse between healthcare specialists and other professionals was out of balance. Their argument was because both parties often possessed information and knowledge that the other party lacked and as a result were incapable of relating to their different viewpoints ( Busari 2012). As a result, discussions between health professionals and specialists’ breakdown too often due to emotionally driven arguments or gaps in information/knowledge. One of the respondents emphasized that trust is essential and that sometimes history of context, dominance, hierarchy and the financial agreements in a department play more significant roles in the process than initially thought. To strive for improvement, the professionals emphasized that determining the right time to initiate leadership education was important but at the same time challenging. Another respondent felt that life experiences and the willingness to take on responsibilities were factors that influenced whether a resident was capable of learning particular leadership skills.

## Conclusion

In summary, the process of teaching in the clinical workplace is subject to ongoing improvement and requires the incorporation of new and different educational approaches such as case-based discussion. The residents and staff we interviewed perceived that the quality of training was influenced strongly by the scarcity of time for teaching. They suggested that more interactive educational strategies like case-based and practice-based education should be adapted into longer and regular protected teaching slots. On leadership education, the need for more focus on trust was emphasized and also considered to be a precondition for any leadership-training program. The respondents observed that mutual trust was lacking between different stakeholders in the health care system and that improving residents’ knowledge of health care systems and the processes involved could help restore this relationship. The findings also suggest that more trust between stakeholders can leverage imbalances in professional interactions and facilitate improved understanding between them. Our results reiterate the challenges associated with finding the perfect moment to initiate formal leadership education in postgraduate medical education. Therefore, finding answers to these challenges and how best to deliver the training in practice would constitute the focus of our ongoing research.

## Take Home Messages

• The process of teaching in the clinical workplace requires the incorporation of new and different educational approaches, like case-based discussion.
• Medical residents and faculty perceive that the quality of training is strongly influenced by the scarcity of time to teach.
• Interactive educational strategies like case-based discussion need to be adopted into regular and protected teaching slots.
• Trust is a prerequisite for good leadership and leadership education
• More research should be conducted to find answers to how leadership training can be delivered effectively in the clinical workplace.

## Notes On Contributors

1. Jamiu O. Busari

Program Director, Pediatric residency program, Department of Pediatrics, Zuyderland Medical Center, Heerlen, Netherlands; Associate Professor, Department of Educational Development and Research, Faculty of Health, Medicine and Life Sciences, University of Maastricht, Maastricht, Netherlands.

2. Lisa N. Isbouts

Medical Resident, Department of Pediatrics, Radboud University Medical Centre, Nijmegen, the Netherlands.
