# Supplementary material for: Can case-based discussions be used for leadership development in medical education? - an exploratory inquiry
Source: MedEdPublish (2016). 2018 Jun 1;7:117. [Version 1] doi: 10.15694/mep.2018.0000117.1 (PMC10699358; doi:10.15694/mep.2018.0000117.1)
Supplement: Peer review history

# Reviewer report: Reviewer response for version 1

**DOI:** 10.21956/mep.19586.r29074

**Published:** 2019-03-14

**Authors:** Richard Hays

**Affiliations:** 1 James Cook University

This review has been migrated. The reviewer awarded 3 stars out of 5 This is an interesting paper that addressses a topical issue. Many in the medical profession are almost obsessed with training students and graduates in leadership, but this is likely to be a complex and not so easy task. The method used was appropriate, although I am not sure about the clarity of the question that was explored and the discussions seemed to cover a lot of territory. To me it is obvious that case-based dis ussions, if constructed appropriately, should be very useful in learning about most things. Why should it be different for leadership? Surely, discussing challenging, illustrative scenarios at the level of principle and sharing experiences, with appropriate facilitation, is much better than learning thru making errors based on inexperience? What came out resembles more a bunch of concerns about workload and time management, rather than what I understood the question to be. Can we increase efficiency by including leadership issues in routine clinical cases that are used in tutorials? I think that would be many opportunities to follow this more integrated approach that places leadership in the context of clinical practice.

Reviewer Expertise:

NA

No decision status is available

# Reviewer report: Reviewer response for version 1

**DOI:** 10.21956/mep.19586.r29075

**Published:** 2018-06-03

**Authors:** P Ravi Shankar

**Affiliations:** 1 American International Medical University

This review has been migrated. The reviewer awarded 4 stars out of 5 This manuscript deals with the role of case-based discussion (CBD) on leadership development among residents. This is a well-written paper with a clear message. The Methods section has been well-described. I would have liked an example of the case/s used for CBD by the authors and how the case addressed the issue of leadership development among residents. As a medical educator, I am all too aware of medical teachers continuing to use teacher-centered approaches despite the increasing body of evidence pointing to the superiority of active learning and learner-centered approaches. I would also be interested in the interview guide used by the authors while carrying out the semi-structured interviews. The conclusions and the take home message are based on the findings of the manuscript. The importance of an understanding of the health system has been stressed by the authors. This may be a challenge in today’s world where medical students and residents may cross international borders for their education as is often the case with students from offshore Caribbean medical schools.

Reviewer Expertise:

NA

No decision status is available
